# Applications of machine learning for simulations of red blood cells in microfluidic devices

**DOI:** 10.1186/s12859-020-3357-5

**Published:** 2020-03-11

**Authors:** Hynek Bachratý, Katarína Bachratá, Michal Chovanec, Iveta Jančigová, Monika Smiešková, Kristína Kovalčíková

**Affiliations:** 1 Department of Software Technologies, Faculty of Management Science and Informatics, University of žilina, Cell-in-fluid Research Group, žilina, Slovakia; 2 Department of Technical Cybernetics, Faculty of Management Science and Informatics, University of žilina, žilina, Slovakia

**Keywords:** Red blood cell, Simulation of fluid, Cell trajectories, Microfluidic device, Neural network

## Abstract

**Background:**

For optimization of microfluidic devices for the analysis of blood samples, it is useful to simulate blood cells as elastic objects in flow of blood plasma. In such numerical models, we primarily need to take into consideration the movement and behavior of the dominant component of the blood, the red blood cells. This can be done quite precisely in small channels and within a short timeframe. However, larger volumes or timescales require different approaches. Instead of simplifying the simulation, we use a neural network to predict the movement of the red blood cells.

**Results:**

The neural network uses data from the numerical simulation for learning, however, the simulation needs only be run once. Alternatively, the data could come from video processing of a recording of a biological experiment. Afterwards, the network is able to predict the movement of the red blood cells because it is a system of bases that gives an approximate cell velocity at each point of the simulation channel as a linear combination of bases.In a simple box geometry, the neural network gives results comparable to predictions using fluid streamlines, however in a channel with obstacles forming slits, the neural network is about five times more accurate.The network can also be used as a discriminator between different situations. We observe about two-fold increase in mean relative error when a network trained on one geometry is used to predict trajectories in a modified geometry. Even larger increase was observed when it was used to predict trajectories of cells with different elastic properties.

**Conclusions:**

While for uncomplicated box channels there is no advantage in using a system of bases instead of a simple prediction using fluid streamlines, in a more complicated geometry, the neural network is significantly more accurate. Another application of this system of bases is using it as a comparison tool for different modeled situations. This has a significant future potential when applied to processing data from videos of microfluidic flows.

## Background

There are currently many open problems in the research of microfluidic devices used for analysis of specific properties of blood samples. Our primary motivation is development and optimization of devices used for efficient circulating tumor cell (CTC) capture, such as described in [1, 2], which can be used for early diagnostics and management of treatment of cancer. The analysis of blood sample properties is just a subset of this overall goal, but nevertheless poses several important questions, such as accurate determination of cell velocities or deformations.

The design, manufacture and testing of a large spectrum of microfluidic devices faces time, technical and financial limits. One of the reasons is that the behavior and dynamics of blood cells in various settings is diverse and complex and to gain quantitative understanding one often needs modeling and simulations of the underlying elasto-mechanical processes. Firstly, the analysis of deformations is a nontrivial problem [3, 4], which cannot be avoided, since the red blood cells (RBCs), the main solid constituent of blood, are primarily characterized by their elasticity. And secondly, various aspects of their dynamics in flow [5–7] also need to be examined.

An inseparable part of the computational approach is an ongoing verification and validation of the models primarily by comparing simulation and biological experiments, such as in [8–10].

In this work, we methodologically generalize this approach. We process and analyze the results of blood flow simulation experiments using machine learning techniques. While such techniques have already been successfully used for detection of red blood cells in images from experiments [11] or for classification of various shapes and states of red blood cells [12, 13], we propose a new direction. We focus on the accurate description of behavior of the fluidic part, the blood plasma, and the immersed elastic RBCs.

We can consider each performed simulation or biological experiment as a source of information capable of describing the general properties of the investigated device and its behavior, which we are going to analyze, compare and optimize. The output from both types of experiments is in the form of a data set that describes the behavior of the RBCs in the device. The behavior is characterized primarily by cell positions, i.e. trajectories, velocities and more generally by cell rotations, inclinations and areas of occurrence [14].

To obtain this kind of data it is necessary to either perform computationally very intensive simulations or non-trivial and technically complicated video processing of usually imperfect recordings of biological experiments that capture the movement of (mostly) red cells. Therefore the goal of this work is to propose a method that extrapolates from this hard-earned description of behavior of specific cells monitored in the experiment. The result is a general and universal description of cell behavior and important properties in the whole channel, including in locations where there were no cells in that particular experiment.

We apply radial basis functions [15, 16] and Kohonen networks [17, 18] that are useful in identification and modeling of non-linear dynamical systems. The output is a universal system of bases of positions and velocities, which allows us to characterize the modeled situation and predict the RBC dynamics. The data source we used was from simulations of flow in a box channel without obstacles and in a channel similar to the one described in [19]. We compared the results to baseline predictions obtained by using tracers (mass points) and fluid streamlines.

## Methods

### Blood flow simulation model

Blood flow simulations at the scale of individual cells typically involve two distinct model parts - the fluid and the cell membrane. These two are coupled and exert influence on one another in the form of forces. Our model uses the lattice-Boltzmann method [20] for the fluid, the spring network model for the cell membrane and a dissipative version of Immersed Boundary Method (DC-IBM) for the coupling.

There are also several particle-based models available (DPD [21], SPH [22]), in which the particles are used to represent not only the elastic membrane, but also the fluid outside and inside of the cell. Another option is to use the Stokes equation with boundary integral methods, which is suitable for investigation of small numbers of cells [6]. However, the lattice-Boltzmann method is often a method of choice for the fluid due to its simplicity and locality, which makes it suitable for parallelization.

In the models that use the lattice-Boltzmann method for the fluid, the IBM is typically used to couple the fluid to the membrane, e.g. in [23]. Unlike these, our coupling is done via a dissipative exchange of forces at the cell membrane nodes:

1 $$ \mathbf{F}_{jf} =\xi(\mathbf{v} - \mathbf{u}),  $$

where **F**_*jf*_ is the sum of all fluid forces acting on the node *j*. The force is proportional to the difference of the velocity **v** of the node (immersed boundary point - IBP) and the fluid velocity **u** at the same position. For the motion of the nodes we use Newton’s equation:

2 $$ m_{IBP}\mathbf{x}^{\prime\prime}_{j} =\mathbf{F}_{j} + \mathbf{F}_{ext},  $$

where **x**_*j*_ is the position of the given node and *m*_*IBP*_ is its mass. Note that **F**_*j*_=**F**_*jf*_+**F**_*je*_, where **F**_*je*_ is the composition of all elastic forces acting on node *j* and **F**_*jf*_ is calculated using Eq. (1). The force **F**_*ext*_ represents the sum of all external forces including those arising from the cell-cell and cell-wall interactions.

**Fig. 1:**
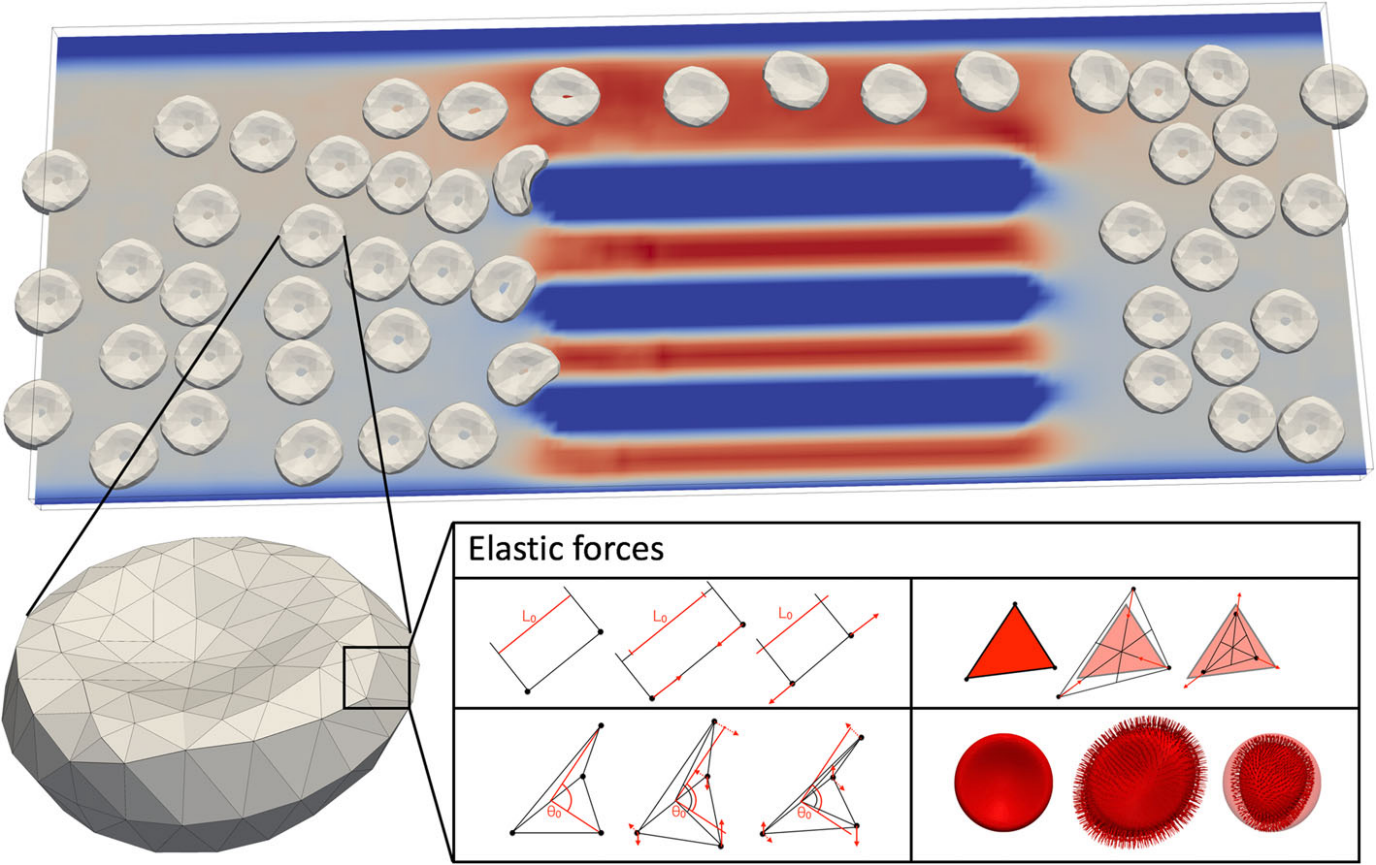
A schematic illustration of the channel with cells. The color represents the fluid velocity (blue for slower and red for faster). Each individual cell is modeled by a spring network of immersed boundary points bound by elastic interactions

For the modeling of elastic properties of cell membrane we use five types of elastic forces. Each one corresponds to one elastic modulus: stretching (preservation of length), bending (preservation of angles between neighboring triangles), conservation of local area, conservation of global area and conservation of volume. A schematic representation of the model is depicted in Fig. 1. The description of implementation can be found in [24] and the current documentation with up-to-date model at [25].

In this simulation model, the following needs to be evaluated at each time step:

- *Local elastic interactions* - If there are *n* nodes (IBPs) representing the cell surface, this means approximately 3*n* evaluations of three local interactions for this cell: stretching, bending and local area.
- *Global elastic interactions* - This amounts to a loop over all *n* nodes to calculate the global surface and volume and then another loop over nodes to apply the global forces to all of them.
- *Cell-wall interactions* - A cell-wall interaction is evaluated for each node that is closer than a predefined cutoff distance to any boundary.
- *Cell-cell interactions* - A cell-cell interaction is evaluated for each pair of nodes belonging to different cells that are closer than a predefined cutoff distance.
- *Cell-fluid interactions* - The forces in Eq. (1) are evaluated for all nodes. This involves a trilinear interpolation of fluid velocity from lattice nodes to IBP position.
- *Movement of IBPs* - For all nodes, the differential equations (2) are solved using the velocity Verlet scheme.
- *Fluid flow* - Multiple-relaxation version of lattice-Boltzmann method is used for propagation and collisions of the density populations in a 3D cubic lattice.

### Simulation setup and parameters

All simulation experiments were performed using the freely-available open-source software ESPREesSo [26] and its LB and Object-in-fluid modules. The surface mesh of red blood cell was generated in Gmsh [27].

We performed two types of simulations for this work. In both of them, the cell was represented by a triangulated mesh with 141 vertices. The numerical parameters of the cell are summarized in Table 1 and the mechanical properties of the fluid are summarized in Table 2.

**Table 1:** Numerical parameters of the cell used in simulations

Parameter	LB units		SI units	
Stretching coefficient *k*_*s*_	7·10^−3^	*LN*/*Lm*	7·10^−6^	*N*/*m*
Bending coefficient *k*_*b*_	2.5·10^−4^	*LNLm*	2.5·10^−19^	*Nm*
Coefficient of local area conservation *k*_*al*_	1·10^−3^	*LN*/*Lm*	1·10^−6^	*N*/*m*
Coefficient of global area conservation *k*_*ag*_	0.9	*LN*/*Lm*	9·10^−4^	*N*/*m*
Coefficient of volume conservation *k*_*v*_	0.5	*LN*/*Lm*^2^	5·10^2^	*N*/*m*^2^
Viscosity of cell membrane *k*_*visc*_	0	*Lm*^2^/*Ls*	0	*m*^2^/*s*

**Table 2:** Numerical parameters of fluid used in simulations

Parameter		LB units			SI units	
LB-grid for fluid		1	*Lm*		1·10^−6^	*m*
Kinematic viscosity		1.5	*Lm*^2^/*Ls*		1.5·10^−6^	*m*^2^/*s*
Density		1	*Lkg*/*Lm*^3^		1·10^3^	*kg*/*m*^3^
Friction coefficient		3.63	[−]		3.63	[−]

There are no predefined units in ESPResSo. For our simulations, we chose a system denoted as an LB-unit system. Values of all variables are indicated in both LB and SI units.

The cell-cell interaction was modelled using the membrane_collision potential with the parameters mc_K = 0.005, mc_n = 2.0, mc_cut = 0.5. The interactions between the cells and the walls and obstacles were modelled using the soft_sphere potential with the parameters soft_K = 0.00035, soft_n = 1.0, soft_cut = 0.5. During the simulation, the position vector of the cell center and the velocity vector of the cell center calculated from positions were saved every 1000 steps, with the simulation step being 0.1 *μs*. Even though the simulation channels were constructed with periodic boundary conditions in the *x*-direction, the output data were processed in order to consider the simulation channel only once and multiple passages of a single cell through the channel were regarded as separate trajectories of different cells.

The two different types of channels, which were used, are labeled A and B.

#### Channel A

The biological experiments described in [19] investigate red blood cell deformability using a flat microfluidic channel with slits of different width. The deformability was assessed by observing cell velocities in different parts of the channel. This enables easier evaluation of cells’ elasticity that has diagnostic use. In order to achieve single-cell precision, the blood was diluted with saline solution in the 1:50 ratio, which represented hematocrit Ht <1%.

Our simulation was run in a periodic channel inspired by these experiments. The channel contained obstacles forming four slits and we used a random initial seeding of 50 cells (Ht=20%). The higher hematocrit was chosen in order to include the cell-cell interactions. The simulation was run twice, each time with a different initial seeding, in order to provide a training and a testing dataset for the neural network.

**Fig. 2:**
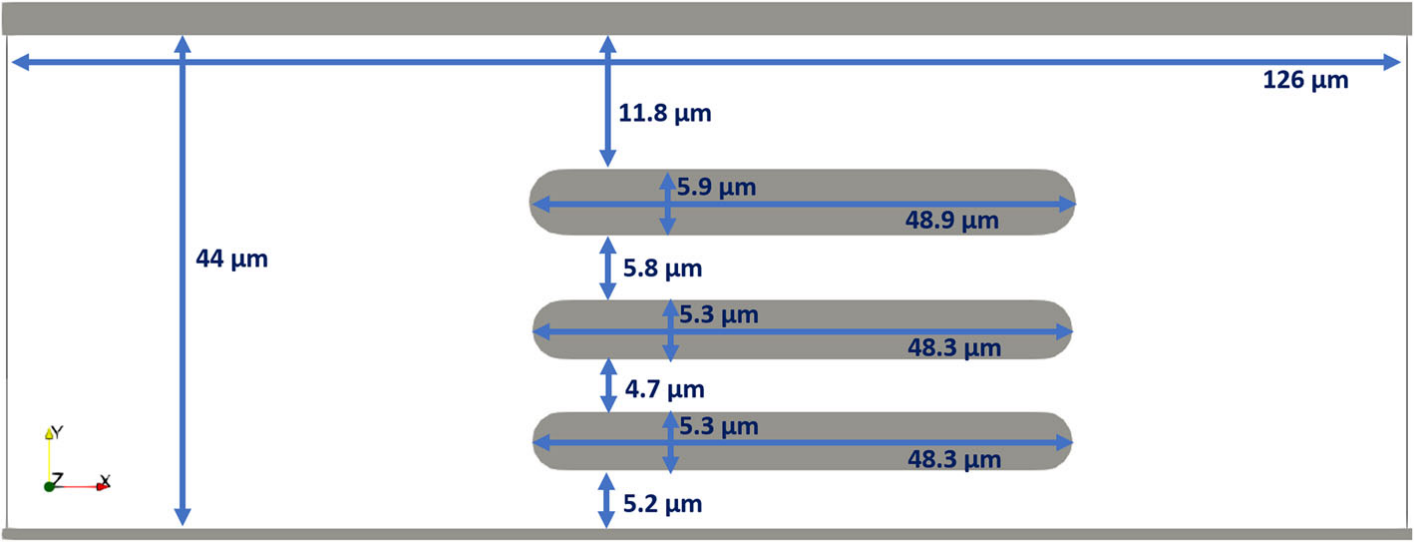
The geometry and dimensions of the simulation channel A. The depth of the channel is 3.5 *μm*

The simulation channel is presented in Fig. 2. The dimensions of the channel were 126.0×44.0×3.5*μm*^3^ and the fluid that carried the cells was flowing in the *x*-direction. This geometry was inspired by the laboratory experiment with RBCs described in [19].

**Fig. 3:**
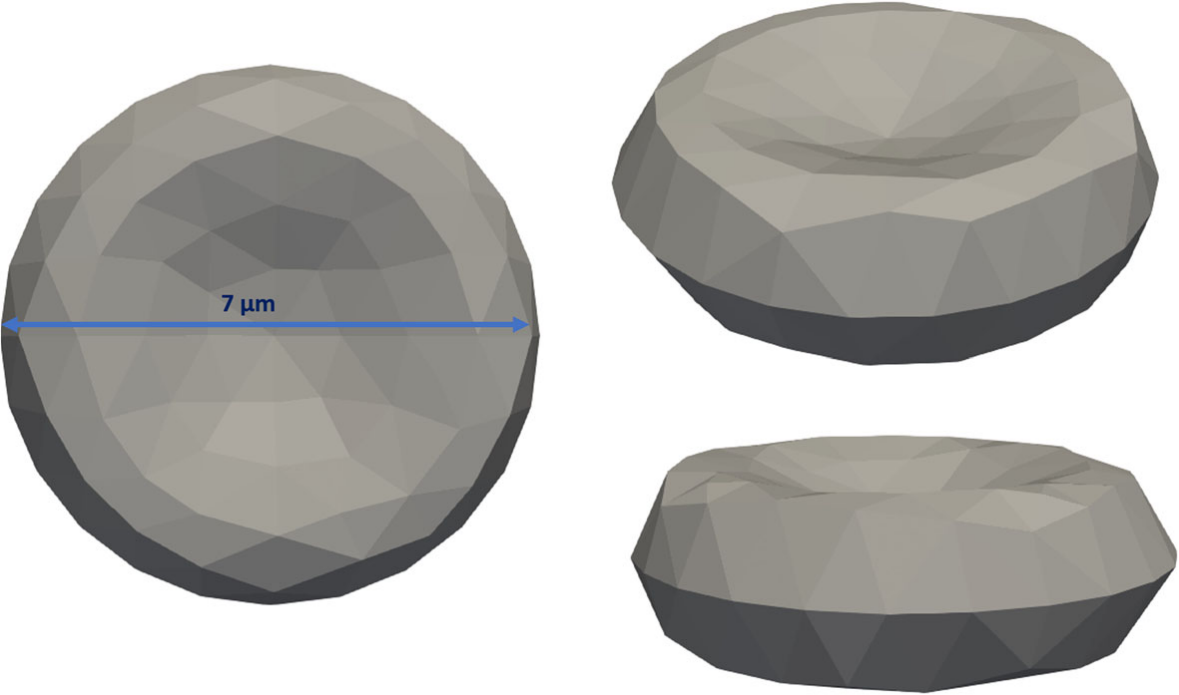
Numerical model of the RBC uses surface discretisation

The model of cells used for the simulation is presented in Fig. 3. The dimensions of each cell were 7.0×7.0×2.3*μm*^3^.

The movement of the fluid in the simulation is caused by an external fluid force, which defines the velocity of the flow inside the channel. These values are presented in Table 3.

**Table 3:** Fluid velocity parameters, channel A

Parameter		LB units			SI units	
External fluid force		6·10^−4^	*LN*		6·10^−13^	*N*
Maximal fluid velocity		5.7·10^−4^	*Lm*/*Ls*		5.7·10^−4^	*m*/*s*

For the cross-validation of the proposed method, two other simulations were run under slightly different conditions. The properties of the liquid were not modified and both simulations were run with 50 randomly seeded cells.

**Fig. 4:**
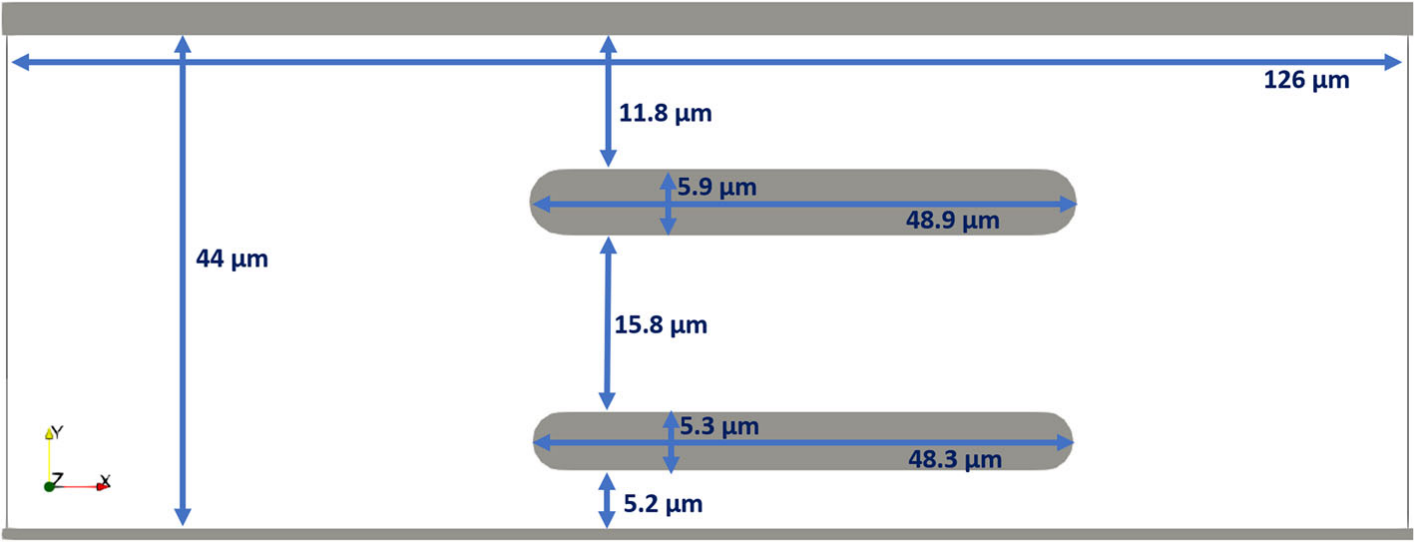
In the modified channel A, the middle obstacle was removed. The depth of the channel remains 3.5 *μm*

The first simulation had a different geometry of the simulation channel. The original channel contained three obstacles, forming four slits. In the modified channel, the middle obstacle was omitted and so there were only three slits, Fig. 4. This corresponds to slightly larger fluid volume and thus slightly lower Ht=19%.

The second simulation had the same geometry and hematocrit as the original channel but the elastic properties of the RBCs were different. The original values of the stretching and the bending elastic coefficients were multiplied by 100 and the original value of the local area conservation coefficient was multiplied by 10. These modifications resulted in significantly stiffer cells. The global area conservation coefficient and the volume conservation coefficient were unchanged.

#### Channel B

**Fig. 5:**
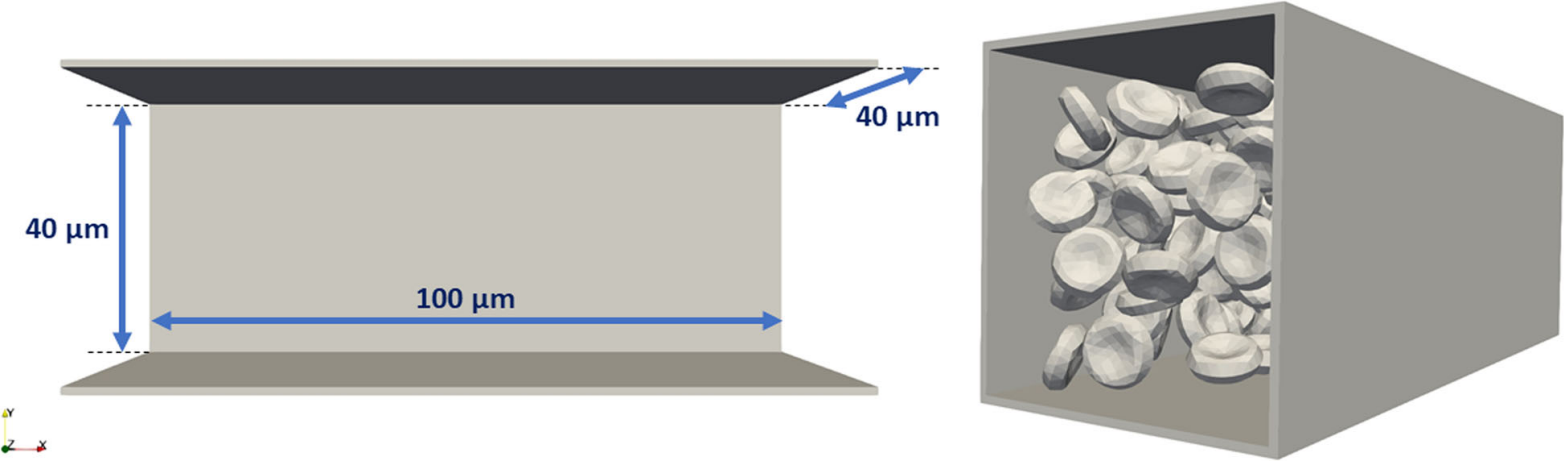
The geometry and dimensions of the simulation channel B

The simulation channel B had a box shape with four walls, which were parallel to the main direction of the flow. The fluid was flowing in the *x*-direction. The dimensions of this simulation box were 100×40×40*μm*^3^. There were no obstacles inside the channel. A precise description of the channel is presented in Fig. 5.

The dimensions of each cell were 7.8×7.8×2.6*μm*^3^. The external fluid force and the corresponding fluid velocity are presented in Table 4. Note that the fluid in this simulation was flowing significantly faster than in channel A.

**Table 4:** Fluid velocity parameters, channel B

Parameter		LB units			SI units	
External fluid force		1·10^−4^	*LN*		1·10^−13^	*N*
Maximal fluid velocity		7.6·10^−3^	*Lm*/*Ls*		7.6·10^−3^	*m*/*s*

For the purpose of this study, we simulated a flow with 177 cells representing approximately 10% hematocrit. These settings were chosen, because channel B is meant as a *baseline* simulation, with no obstacles, uniform velocity and infrequent cell-cell interactions. Similarly to the first channel, the simulation was run twice, with two different initial seedings, in order to provide a training and a testing dataset for the neural network.

In the following, we describe the results obtained by analyzing and comparing several simulations. Table 5 lists their notation and basic parameters. The initial seeding of cells was random and unique for every simulation.

**Table 5:** Notation used for simulations with respect to geometry, number of cells and seeding

Simulation ID	Channel	Description	Seeding	No. of cells	Dataset
A50a	A	normal	a	50	training
A50b	A	normal	b	50	testing
A50c	A	stiff RBC	c	50	testing
A50d	A	new geometry	d	50	testing
B177e	B	no obstacles	e	177	training
B177f	B	no obstacles	f	177	testing

### Machine learning algorithms

In this section, we describe a method that we used to create a system of bases of velocities in specific channels. Using this system, we can then predict the cell velocities and consequently, the cell movement. We obtain the system of bases using a neural network with unsupervised learning inspired by the radial basis function network and self-organized Kohonen maps.

The system of bases obtained this way characterizes the microfluidic channel and can also be used for comparison. Two different seedings using the same model and the same channel resemble one another more than two different channels or two different cell models. Another use case, the one we focus on in this work, is the prediction of cell trajectories using the velocities obtained from linear combination of bases.

#### Overview

One of the areas for which the neural networks are very suitable is the approximation of functions. The concept of approximation as a linear combination of special non-linear functions is known, for example, from the field of signal processing, where the basis functions are the Shannon function and its shifts in time [28].

For radial basis function networks, non-linear functions with the property that they depend only on the size of the input vector **x**, or possibly on its distance from a certain fixed center **c**, are used as basis functions. A Gauss function is a typical example of a radial basis function:

3 $$ \varphi\left(\mathbf{x}, \mathbf{c}\right) = e^{-\left(\alpha\cdot \sqrt{\sum\limits_{k=1}^{N}({x_{k}}-{c_{k}})^{2}}\right)^{2}}.  $$

The necessary decomposition coefficients can be found using a neural network.

A self-organizing map (SOM), introduced by Kohonen [17, 18], uses not only weights for the individual neurons, but also considers their geometric location using a neighborhood function. During the learning phase, the weights of SOM reflect the statistical properties of the values of input vectors from the training set.

A specific property of SOM is that it can use the topology and display the characteristic features of the training data set as they relate to the data position. This is the reason why the SOM neurons are organized in a regular structure (lattice or list). The output is geometrically sorted and thus it is possible to find a neighbor.

Consequently, an assumption for learning is that the neighboring neurons have similar values and more distant neurons have more different values. One can take advantage of this by using the Euclidian distance and setting the value 1 (representing the radius of the important neighborhood) properly. After squaring, values smaller than 1 decrease and values larger than 1 increase. This way one can influence, which values have more and which less weight in the learning process when creating the system of bases.

#### The learning process of the system of bases

Compared to the videos from biological experiments, using data from simulations for the learning process has the advantage that we can include many more details of the cell dynamics. Image processing of videos of cell flows typically results in bounding boxes or approximate 2D cell shapes, their centers and velocities. In cell-scale simulations, in addition to these characteristics, we can also observe the full 3D shape, cell rotations, local velocities at different parts of the membrane and more. From this perspective, it is more suitable to examine the quality of machine learning predictions by training and testing them on various simulation data. Once they work satisfactorily, they can be applied to data extracted from images.

For this work, we used the two training sets (one for each considered channel, A and B) as the input for the learning algorithm, which contained information about the cell center positions and velocities in simulation experiments. In simulation A50a center position and velocity information about 50 randomly seeded cells were recorded 15 430 times (every 1 000 simulation steps). This comprises 771 500 data vectors for the training set. In simulation B177e, we recorded the center trajectories and velocity vectors for 177 randomly seeded RBCs cells 2 269 times (every 1 000 simulation steps). This means 401 613 data vectors as a training set.

We obtained the system of bases by unsupervised neural network learning inspired by the previously mentioned radial basis function network and self-organizing Kohonen maps. We used *N*=256, *N*=1 024, *N*=4 056 and *N*=8 112 basis vectors in the neural networks. In the following, we present the results of the analysis using the *N*=8 112 basis vectors.

To initialize the system of bases **B**, we used the output of simulation experiments. Firstly, we selected the initial system **B**^0^ that consists of six-component vectors $B_{i}^{0}(\mathbf {a}, \mathbf {b})$ for *i*∈{1,2,…*N*}, which represent positions $B_{i}^{0}(\mathbf {a})$ and corresponding velocities $B_{i}^{0}(\mathbf {b})$. Typically, this selection is random, taking the input vectors (**x**,**y**) of positions and velocities from the training set. The benefit of this approach is that fewer time steps and fewer vectors in the system of bases are needed for the neural network training compared to completely random initialisation.

The initialized system of basis vectors **B**^0^ was then iterated using input vectors (**x**,**y**) randomly selected from the training set. For the training, we have chosen the following radial function as the similarity function for comparing the basis *B*_*i*_(**a**) to the position **x** from the training set:

4 $$ s_{i}(\mathbf{x}) = e^{-k \,\cdot {||{\mathbf{x}}-{B_{i}(\mathbf{a})}||^{2}_{2}} },  $$

where *k*>0 is a shape constant, which determines how steep the basis is. We used the value *k*=10 for simulations in channel A and *k*=1 for simulations in channel B. Note that the values of the similarity function *s*_*i*_(**x**) are from the interval (0,1〉.

The approach to training of the basis vectors is adopted from the Kohonen networks concepts. For an input vector (**x**,**y**) we found the closest basis, *B*_*max*_, which is the one with the largest value *s*_*i*_. We then adjusted the position of *B*_*max*_ in the next step. The iterated basis position was determined using the following formula:

5 $$ B_{max}^{n+1}(\mathbf{a}) = B_{max}^{n}(\mathbf{a}) + \eta(\mathbf{x} - B_{max}^{n}(\mathbf{a})),  $$

where *η* is the learning rate. We used the value *η*=0.1.

For all other basis vectors *B*_*oth*_ we had

6 $$ B_{oth}^{n+1}(\mathbf{a}) = B_{oth}^{n}(\mathbf{a}).  $$

The advantage of this approach over the back-propagation algorithm is that it needs a smaller dataset and shorter learning time.

For the next calculations, we normalized the values of the similarity function *s*_*i*_(**x**) so that their sum *s*(**x**)=1:

7 $$ s_{i}' (\mathbf{x}) = \frac{s_{i} (\mathbf{x})}{\sum\limits_{i = 1}^{N} s_{i} (\mathbf{x})}.  $$

The predicted velocity at position **x** is then a linear combination of $ B_{i}^{n}(\mathbf {b})$ with coefficients $s_{i}^{\prime } (\mathbf {x})$:

8 $$ \mathbf{y}^{\prime} (\mathbf{x})= \sum_{i = 1}^{N} s_{i}^{\prime} (\mathbf{x})\,\cdot B_{i}^{n}(\mathbf{b}).  $$

To determine the velocities in the system of bases, i.e. to determine the components *B*_*i*_(**b**), we used the stochastic gradient descent method to get a system of bases **B**={*B*_*i*_(**a**,**b**),*i*=1...*N*}, which minimizes the error *E*=**y**−**y**^′^(**x**). For calculation of the iterated velocity values $B_{i}^{n+1} (\mathbf {b})$, we used the formula:

9 $$ B_{i}^{n+1} (\mathbf{b}) = B_{i}^{n} (\mathbf{b}) + \eta\left(\mathbf{y}-\sum_{i = 1}^{N} s_{i}^{\prime} (\mathbf{x})\,\cdot B_{i}^{n}(\mathbf{b})\right)\cdot s_{i}^{\prime} (\mathbf{x}).  $$

Due to the small depth of the network, the learning time was very short and we did not need a large dataset. The network weights were fully trained (with respect to *η* and the size of the data set) after five training epochs. The number of iterations was 4·10^6^ for channel A and 6·10^6^ for channel B. In each iteration, a random sample was selected from the data set and the network weights were adjusted. Using an i7-7700k processor, the training took about 10 minutes.

#### Prediction of velocities using the system of bases

Once the neural network was trained, we got a system of basis vectors *B*_*i*_(**a**,**b**). This system can be used for prediction of velocities of RBCs at positions **x** using the formula

10 $$ \mathbf{y} (\mathbf{x})= \sum_{i = 1}^{N} s_{i}^{\prime} (\mathbf{x})\,\cdot B_{i}(\mathbf{b}),  $$

which is analogous to Eq. (8) that was used for iterations of velocity components in the system of bases.

## Results

To validate the system of bases generated for the two channels, we used several methods that correspond to some of the expected use cases regarding the RBC velocity predictions. In the following, we describe some of the validation approaches and measurement of the prediction accuracy of system of bases trained using the training dataset when applied to the testing dataset.

### Cross validation simulations

We run different simulations for the channel A. In addition to the original simulation A50a, we also performed a simulation with stiffer RBCs (A50c) and a simulation with modified channel topology (A50d). We then used the A50a simulation as the training set and the A50c and A50d simulations as the testing datasets. We expected worse prediction results for both of these cases.

More specifically, we removed the central obstacle in simulation A50d. We then observed the effect of faster flow of cells that do not slow down due to the contact with obstacle. Also, the cells passing this part of the channel now do not need to deform to pass through the missing 4.7 and 5.8 *μm* slits but can travel through an essentially free 15.8 *μm* corridor instead.

In the simulation A50c, we examined even more dramatic changes. Artificially stiff RBCs can only pass the 5.8 *μm* slit between obstacles and even this they do significantly more slowly. They cannot deform to pass the two narrower slits and thus they block them and accumulate around the entries.

### Local velocity prediction error

For the evaluation of accuracy of velocity prediction, we used the RBC testing data in the format (**x**,**y**), where **x** represents the position of the center and **y** its velocity vector. We neglected their mutual relations, i.e. the fact that the cells are following trajectories, and considered each data vector as a purely local information about the velocity of the RBC center at the given location. We calculated the predicted velocity vector **y**_*p*_ for position **x** using Eq. (10). We then evaluated how close this calculated prediction **y**_*p*_ is to the corresponding velocity **y**.

In order to do that, we measured the difference of the velocities as a percentage of the deviation from the expected value of the velocity **y**. For each data vector (**x**,**y**) we obtained a dimensionless relative error $RE_{i} = \frac {||\mathbf {y}- \mathbf {y}_{p}||_{2}}{ ||\mathbf {y}||_{2}} \cdot 100 [\%]$. The number of the testing vectors (**x**,**y**) for individual tests are listed in Table 6 together with the respective mean relative error (MRE).

**Table 6:** The dimensionless MRE (in %) for velocity predictions

Training set	A50a	B177e	A50a	A50a
Testing set	A50b	B177f	A50d	A50c
No. of compared velocities	309816	473154	706794	704769
MRE	11.9	8.9	21.0	468.5
MRE for top 99.9% of results	11.8	8.9	20.8	442.1
MRE for top 99% of results	11.3	8.6	20.2	352.5
MRE for top 90% of results	9.8	6.9	17.6	52.1
MRE for top 50% of results	6.1	3.2	10.1	6.6

In Table 6, we also report similar results calculated after leaving out 0.1*%*, 1%, 10% or 50% of the data with the largest difference ||**y**−**y**_*p*_||. These data can be considered as a specific form of outliers where the value of **y** is influenced by the numerical fluctuations caused by the short-term oscillations of the numerical model of the surface of the RBC when passing through the critical points of the channel.

The first two columns of Table 6 document the MRE for the original training and testing datasets in both channels. As expected, we observe smaller errors for the simpler and more predictable channel B.

In the validation columns, we observe the expected increase in errors. For the simulation A50d, we see approximately doubling of the error of velocity prediction. In the case of simulation A50c with the stiff cells and two blocked slits, the increase in the error is significantly larger. Interestingly, the error value for top half of the cells, those which travel through the wide part of the channel, confirms that this part of flow is still similar to the baseline simulation and the stiff cells pass it in an almost identical manner.

### Global trajectory prediction error

In order to measure the precision of the neural network velocity prediction, we also consider the whole trajectories of the individual RBCs in the simulation experiment. We compare these simulated (required) trajectories with the predicted trajectories that start from the same positions. The predicted trajectories are obtained by numerical integration of cell motion using the predicted velocities. Therefore for each cell *i*, its initial position, $\mathbf {x}_{0}^{i}$, is identical with the initial position of the cell in the simulation and its further motion is determined by the formula

11 $$ \mathbf{x}_{n+1}^{i} = \mathbf{x}_{n}^{i} + \mathbf{y}(\mathbf{x}_{n}^{i}) \cdot dt,  $$

where $\mathbf {y}(\mathbf {x}^{i}_{n})$ is the velocity, calculated using Eq. (10), and with respect to the chosen unit system, the value of the *dt* is 1.

For each cell, the number of iterations, and thus positions generated for the predicted trajectory, matches the number of positions recorded for the simulated trajectory. This way we get a pair of corresponding trajectories, for which we can compare the RBC center positions and calculate their distance in *μm*.

**Fig. 6:**
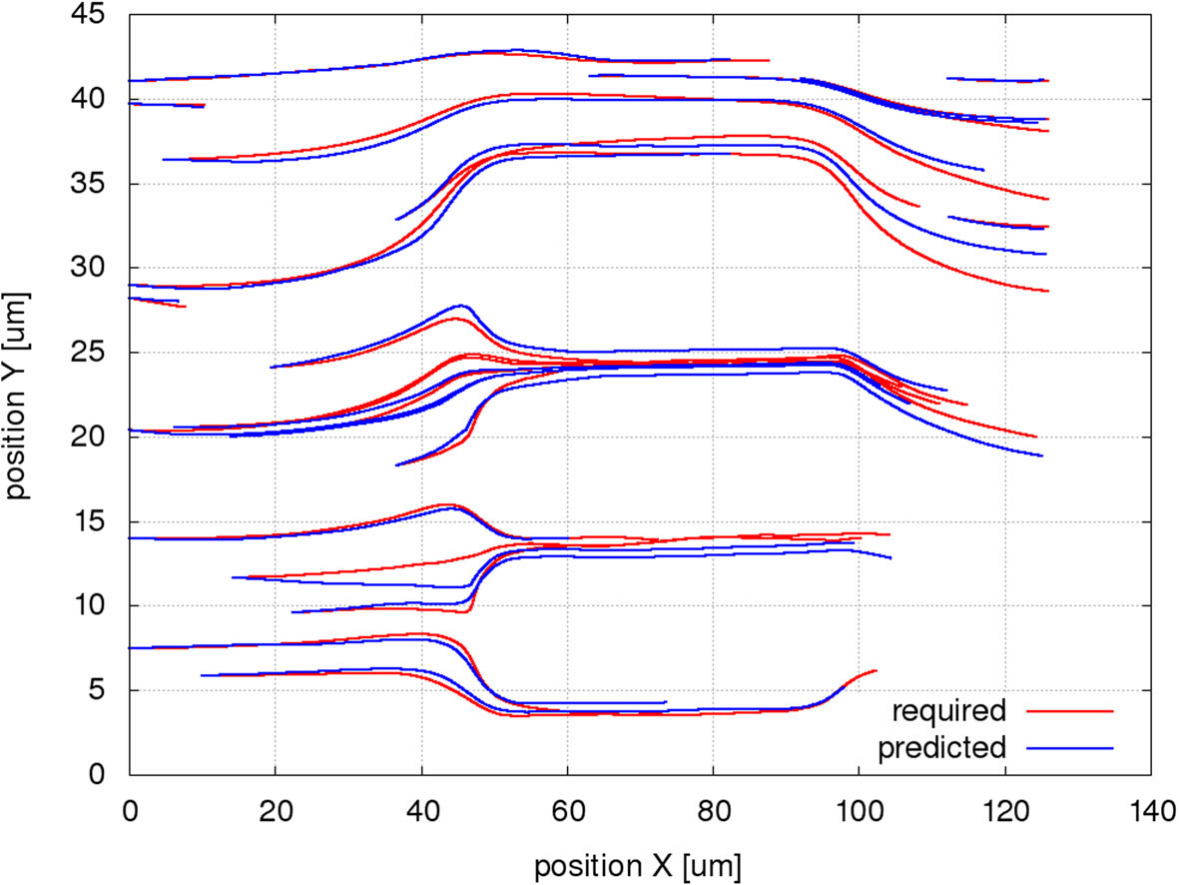
Simulated (required) and predicted trajectories for a selection of 20 RBCs in channel A. The simulated trajectories (red) are taken from the simulation A50b, the predicted trajectories (blue) are calculated using the system of bases trained on the simulation A50a

**Fig. 7:**
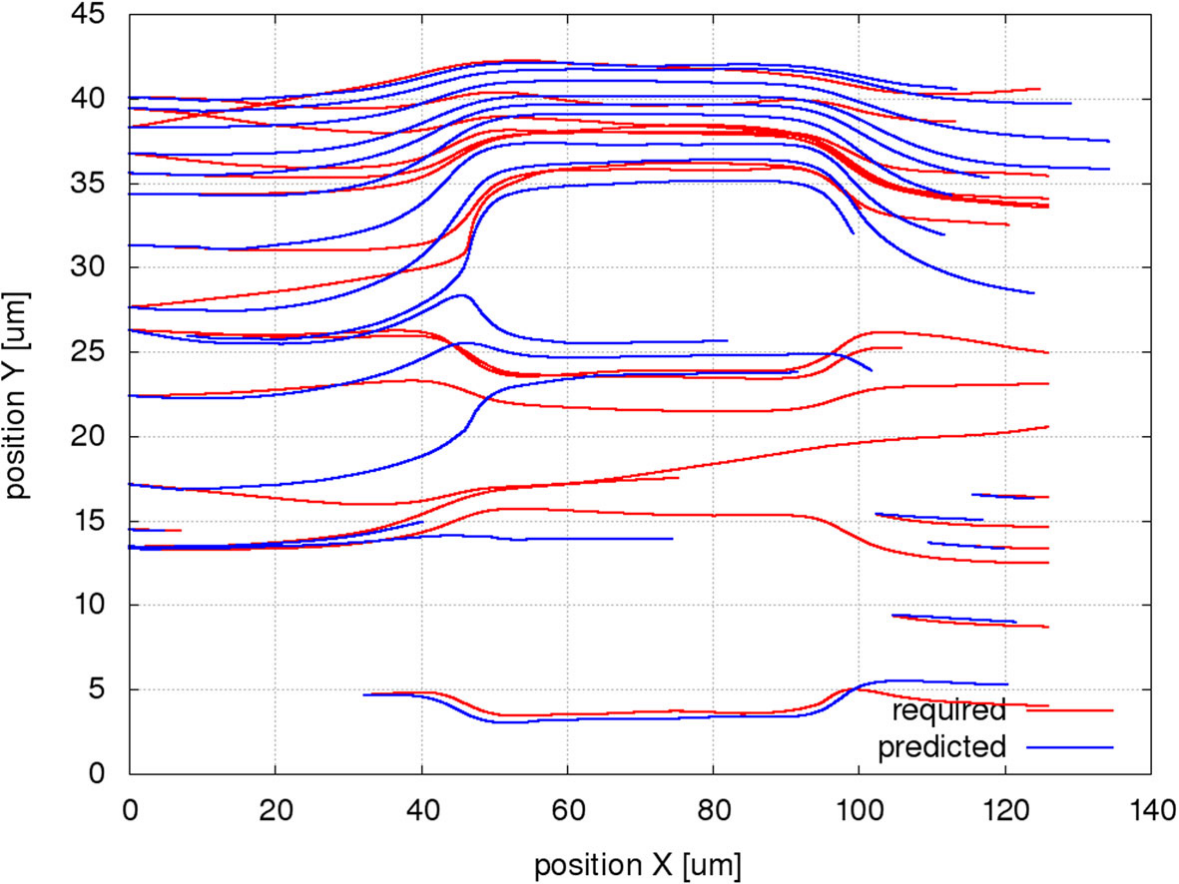
Simulated (required) and predicted trajectories for a selection of 20 RBCs in a modified channel A. The simulated trajectories (red) are taken from simulation A50d (middle obstacle removed) and the predicted trajectories starting from the same positions are calculated using the system of bases trained on the simulation A50a (channel with all obstacles). The simulated trajectories use also the space freed by the obstacle while the predicted trajectories enter the slits of the original channel

**Fig. 8:**
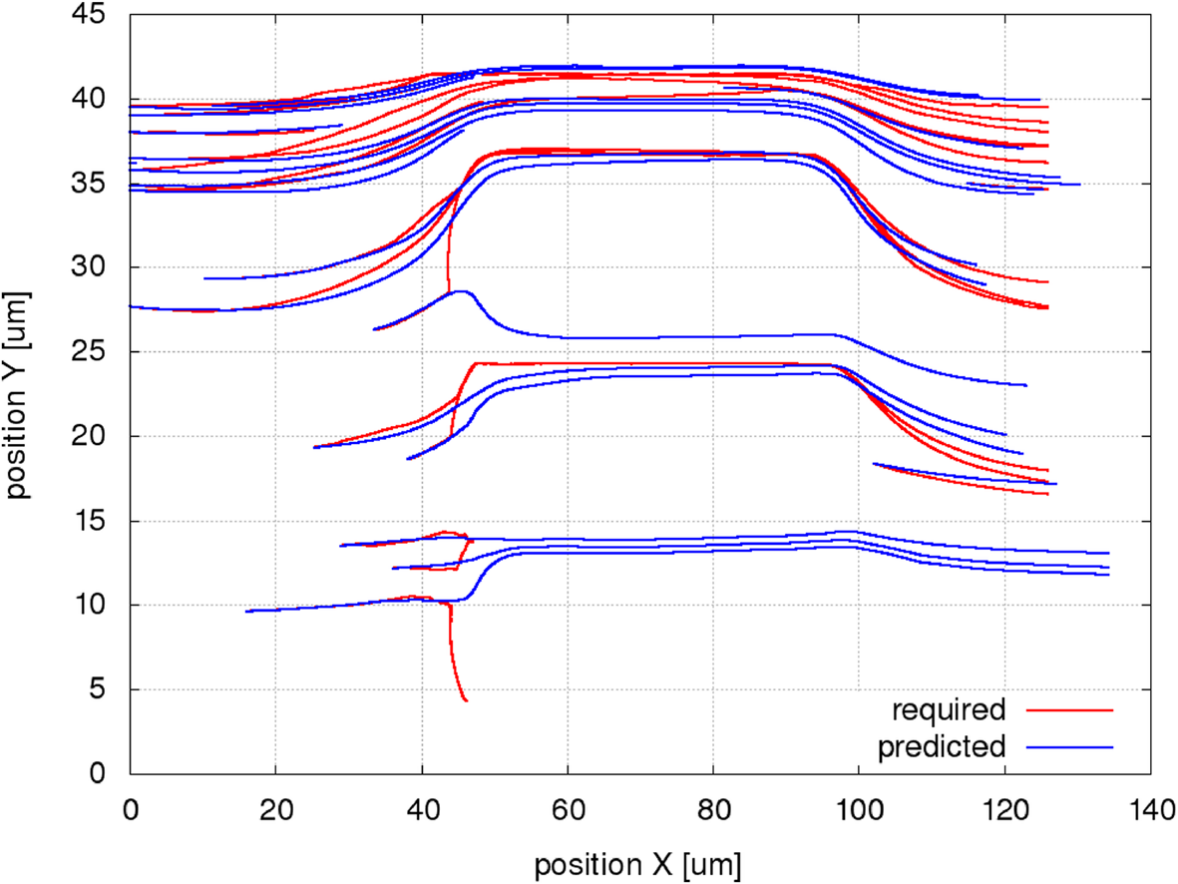
Simulated (required) and predicted trajectories for a selection of 20 RBCs in a simulation with stiff cells (channel A). The simulated trajectories (red) are taken from simulation A50c. As we can see, the stiff RBCs are unable to pass through the narrowest slits of the channel and block it. The predicted trajectories were calculated using the system of bases trained on the simulation A50a, in which the regular RBCs moved fluently through all the slits of the original channel

Figures 6, 7 and 8 present the simulated and the predicted trajectories for a selection of 20 RBCs in the channel A for the original setup and for the two modifications (different geometry, different cell stiffness).

**Fig. 9:**
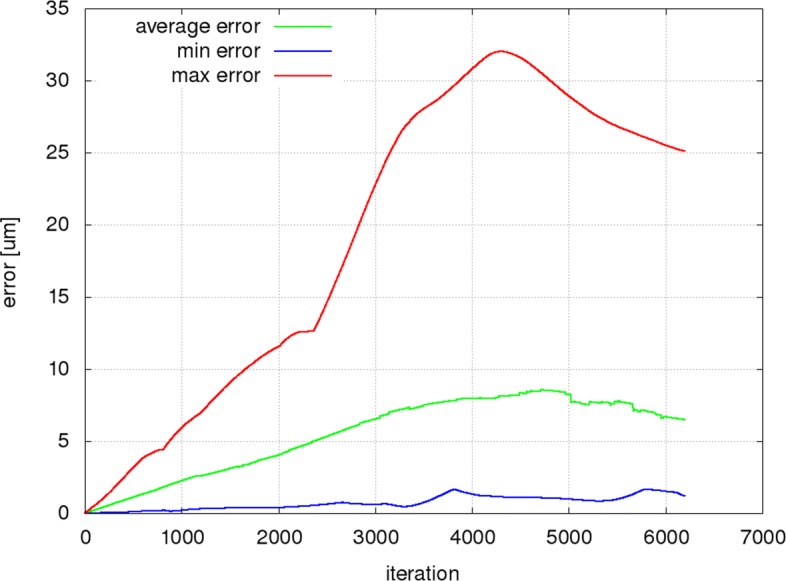
Prediction error is calculated as the distance of RBC centers for all simulated cells from A50b and centers predicted by a system of bases trained on A50a. For the first 1000 iterations, the average error is less than 2.3 *μ*m, corresponding to about 2% of the channel length and 1/3 of the RBC diameter. In the first 2000 iterations, the average error does not exceed 3% of the channel length and half of the RBC size. The highest average error is at the order of the cell size

**Fig. 10:**
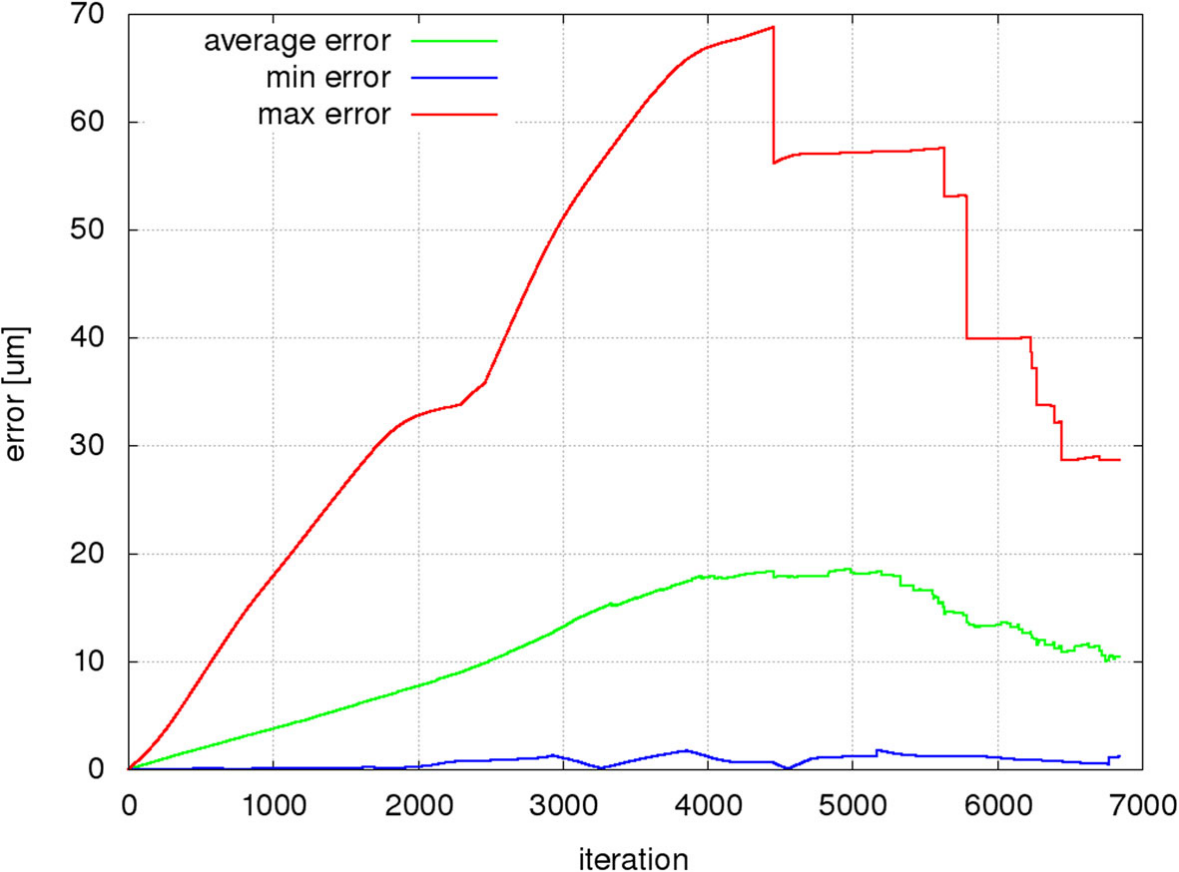
The removal of middle obstacle affects the trajectory differences. The prediction errors for the modified channel have risen approximately two times compared to the errors in Figure 9. After the first 1000 iterations, the average error value is 166% and after the first 2000 iterations 191% of the corresponding values in Fig. 9

**Fig. 11:**
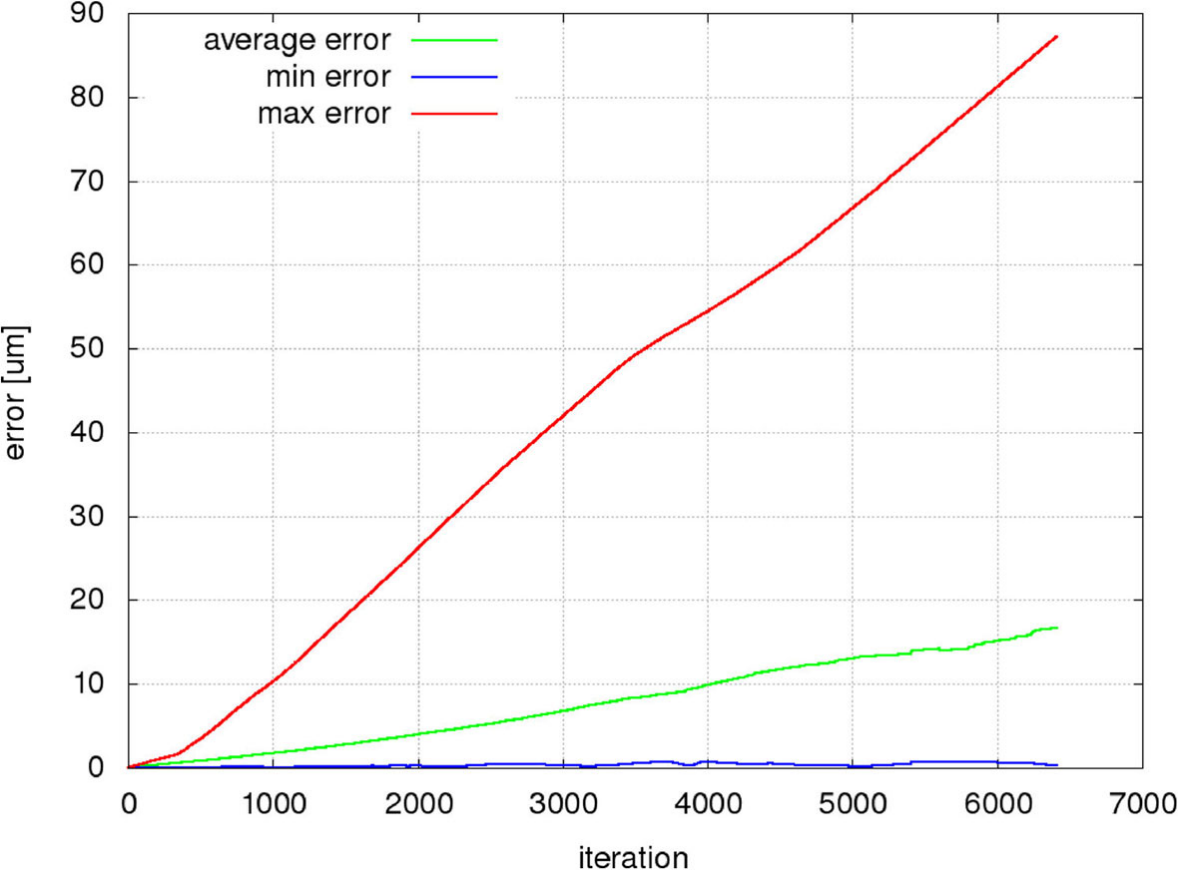
Changing the cell stiffness has a surprising effect on the trajectory differences. Due to the fact that the majority of cells in the channel passage utilise the sufficiently wide slit on the top of the channel, the average error value is very similar to the baseline comparison of simulations A50a and A50b. The differences in the simulations are reflected in the maximum error. Its rapid linear growth corresponds to the deviation of the predicted trajectories from the simulated trajectories of the stuck RBCs

**Fig. 12:**
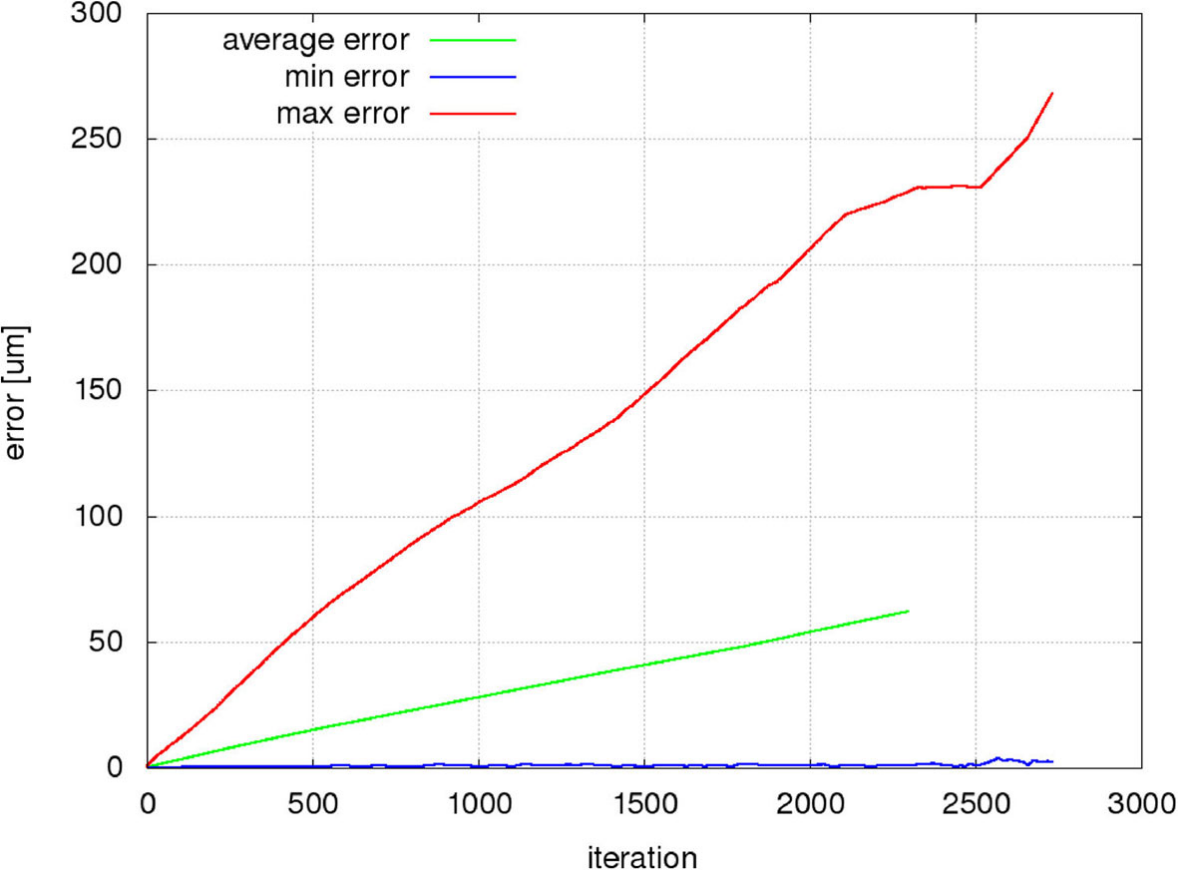
Prediction error calculated as the distance of RBC centers from simulation B177f and centers predicted by a system of bases trained on B177e. The error values are large at first glance. This is due to the fact that the RBCs in channel B are moving more than 20 times faster than the cells in the channel A. If we reduce the errors 20-fold, they reach 50–60% of the error values in Fig. 9

Figures 9, 10 and 11 present the mean, minimum and maximum deviation (error) of the RBC center positions for each pair of trajectories in the three individual experiments as a function of the number of iterations. Figure 12 shows the mean, minimum and maximum deviation (error) for channel B.

### Comparison to predictions using fluid streamlines

A simplified modeling approach is to simulate the cell movement as movement of tracers - points following the fluid streamlines. While such results are less accurate than full simulations with elastic cells, they can also be used as predictions of cell movement. We compared the predictions given by neural networks with the predictions obtained using fluid streamlines.

The fluid information is available only in the lattice nodes and therefore a trilinear interpolation was used to obtain the fluid velocity at the positions of the cell centers.

Every 1000 time steps (100 *μs*), a prediction

12 $$ \mathbf{x}_{p} = \mathbf{x}_{c} + 100\mathbf{y}_{i}  $$

was made for each cell, where **x**_*p*_ is the predicted position, **x**_*c*_ is the current position and **y**_*i*_ is the interpolated cell velocity calculated from fluid velocity.

It is possible to calculate the error in position from known values as ||**x**−**x**_*p*_||_2_, where **x** is the actual position of the cell. Then we calculate the mean error $\bar {\mathbf {e}}$ over all cells in the simulation A as

13 $$ \bar{\mathbf{e}} = \frac{1}{n_{cells}}\sum_{i=1}^{n_{cells}}||\mathbf{x}^{i} - \mathbf{x}_{p}^{i}||_{2}.  $$

Intuitively, this number can be interpreted as distance (in *μm*), which represents a radius defining a ball around the predicted position, where we can reasonably expect the cell center in the next time step.

In the channel A, the fictitious cells moving along the trajectories predicted by the fluid streamlines travel significantly faster than the simulated cells. This is caused by the fact that the channel is flat, only few micrometers high, with four fluid discretization points in the *z*-direction. Due to the parabolic flow profile, the fluid velocity in the center of the channel, which is taken for the cell velocity when making the prediction, is higher than the velocity 1*μm* above or below the center – these are the approximate *z*-coordinates of the majority of cell nodes. Consequently, the simulated cells which *feel* the fluid in these IBPs are effectively carried by smaller velocities.

To approximate this effect, we calculated the predicted cell velocity by weighing three different fluid velocities according to the number of membrane nodes with the given *z*-coordinates. We checked how many of the 141 cell membrane nodes were closer to the center than to the positions offset by 1 (30 nodes) and weighted the maximum velocity accordingly (by a factor 30/141). The remaining 111 nodes were used to equally weigh the upper and lower velocities (by a factor 55.5/141 each). These are approximations, since the actual *z*-coordinate of all nodes varies during the simulation, but they are reasonably precise on the average:

14 $$ \mathbf{y}^{i} = \frac{55.5 \mathbf{y}_{u}^{i} + 30 \mathbf{y}_{c}^{i} + 55.5 \mathbf{y}_{l}^{i}}{141},  $$

where **y**^*i*^ is the approximated cell velocity used in Eq. (12), $\mathbf {y}_{c}^{i}$ is the fluid velocity at the cell center [*x*,*y*,*z*], $\mathbf {y}_{u}^{i}$ is the fluid velocity at [*x*,*y*,*z*+1] and $\mathbf {y}_{l}^{i}$ is the fluid velocity at [*x*,*y*,*z*−1]. The weights add up to 141, which corresponds to the 141 nodes used for discretization of the cell membrane. The predicted trajectories were then calculated using these velocities.

This method is still imperfect, with large mean error $\bar {\mathbf {e}} = 0.009$, which represents about 60% relative error (with respect to the average distance covered by a cell over the same time period, which was 0.015 *μm*) and suggests that one should look for alternative approaches in nontrivial cases like this one. The positions calculated from velocities obtained using a system of bases (A50a) give average error $\bar {\mathbf {e}} = 0.00183$, which represents about 12.2% relative error that is much better.

For comparison, in the box channel B, with 177 cells and no obstacles, the mean local prediction error using streamlines is $\bar {\mathbf {e}} = 0.0166$, which represents about 4% relative error (with respect to the average distance covered by a cell: 0.42 *μm*).

## Discussion

The output of the neural network is a system of bases **B**. Some of the computational time and resources saved in the process of its creation (compared to performing extensive replications and modifications of model simulations) are then used for fast and computationally easy calculation of required information. Here we discuss the basic possible use cases of this approach.

### Local prediction

Using Eq. (10), we can calculate the predicted velocity of the RBC center **y** at any position **x** of the microfluidic device using the system of bases **B**. This prediction allows us to determine and use the RBC velocity even at positions where there were no blood cells in the simulation or where we cannot obtain information from experimental data. Consider also the important opposite situation, in which the velocity predicted by the neural network includes and cumulates the information about movement of multiple cells passing through a given position and its vicinity and thus determines the typical velocity value at this location.

The information about velocity helps to answer some questions when examining properties of a given channel or simulation, such as expected frequency of RBC-rare cells collisions near obstacles used for rare cells capture [29] or estimate of rare cell velocity when it is carried by a stream of nearby RBCs. The cell velocity at various places in the channel may also be important when considering the possible cell damage [30] or serve as a proxy when identifying cell types using narrow parts of channels [31].

### Prediction of cell trajectory

The predicted velocity of RBC center at a given position can be used for calculation of the center position (and thus RBC position) in the next time step. In our case, we chose to use the time step equal to the time step, in which we obtained the RBC data (which was 1000 times coarser than the simulation time step). It is possible though to use a finer time step when necessary.

By repeating this calculation, it is possible to use the neural network to create a trajectory of a fictitious cell from an arbitrary position. Short trajectory segments can be predicted with a good accuracy and thus be used for tracking specific RBCs during video processing of biological experiments. It is a very useful information since especially in higher hematocrits one often encounters cell overlap and after the subsequent separation, the cells have to be re-identified and broken trajectories connected.

The ability to predict cell positions at larger distances and times can improve the efficiency and accuracy of the tracking algorithms. The neural network for such predictions can be trained either on a simulation experiment if we can reasonably ensure its correspondence to the biological experiment or on data from partial video processing, where the cell movement data come from the non-problematic time sequences of the video recordings [32].

### Generation of artificial datasets

If we increase the length of the predicted trajectory, we get less precise predictions, but we can examine the properties of cell trajectories even in locations, where we do not have simulation data. If needed, we can generate an artificial RBC trajectory through the whole microfluidic device. Consequently, by creating such trajectories for a new seeding of sufficiently many RBCs, we can create a replication of a simulation experiment. The computational time needed to obtain such results using the prediction abilities of the system of bases is at least two orders of magnitude shorter than the time needed to perform a new simulation experiment. The computational times, including the training of the system of bases for the experiments described in this work, did not exceed few tens of minutes.

### Characterization of channel and simulation

The trained system of bases can be also considered as a certain description of global properties of a simulation experiment or microfluidic device, in which the experiment was performed. In [32] we can see several results confirming this statement. Comparison of parameters and properties of neural networks trained on data from both computational and biological experiments can serve as an efficient tool for verification of consistency of these experiments.

### Extension to other types of data

The applications mentioned so far considered a system of bases for a position of the RBC center and its velocity. Using a similar approach described in [14] and [33], it is possible to obtain data about other RBC characteristics, which describe the general cell properties, e.g. rotation and inclination, or more specific properties, e.g. periodicity in velocity, rate of deformation, in the individual experiments.

Including these types of data significantly expands the capabilities of this approach, while from the formal, mathematical point of view it is just a different interpretation of a system of vectors, which serve as the input and output of the neural networks. The proposed learning method for the system of bases can be almost mechanically transferred to other, more specialized data.

## Conclusions

Many problems are waiting for extreme-scale high performance computer systems. Harnessing the power of machine learning and starting to answer some of these questions might prove a more accessible path. Neural networks have already been successfully used for cell detection in images and for cell classification of shapes of healthy cells, for recognizing the infected cells among the healthy cells and in a few other problems. Our work shows a new application that concerns the characteristics of flow.

The system of bases can be used for predictions of cell trajectories. This is helpful in several ways. Firstly, for image processing, since the recordings are often imperfect, and trajectories constructed from detected cells are broken into several parts due to undetected cells in some frames (due to cell-overlap or various other reasons. Predictions can be used to correctly connect the fragmented trajectories. The predictions can also help create new data, since after training they can successfully predict a trajectory of a new *fictitious* cell that was not part of experiment, at arbitrary position in the channel.

The system of bases can also be used to efficiently create new datasets with the same characteristics as the simulated computational experiments or image-processed biological experiments. This can be useful for further statistical analysis.

Moreover, comparing parameters and properties of networks trained on data from different experiments (be they computational or biological) can help us distinguish between them. This can be useful for determining how well the simulated model corresponds to the experiment, but also to check for inconsistencies among the experiments themselves.

While this work focused on position and velocity data, it can be easily extended to other types of data and thus be useful tool in elucidating the processes happening in blood flow.

## Data Availability

The simulations were performed using ESPResSo http://espressomd.org. The data from simulations and source codes for the SOMs are available at https://github.com/michalnand/ml\_basis\_sim\_rbc.

## Abbreviations

CTC: Circulating tumor cell
DC-IBM: Dissipative coupling version of immersed boundary method
DPD: Dissipative particle dynamics
ESPResSo: Extensible simulation package for research on soft matter
Ht: Hematocrit
IBPs: Immersed boundary points
LB: lattice-Boltzmann
MRE: Mean relative error
RBCs: Red blood cells
RE: Relative error
SI: Systeme internationale
SOM: Self-organizing map
SPH: Smoothed particle hydrodynamics
